# Assessing Time Series Reversibility through Permutation Patterns

**DOI:** 10.3390/e20090665

**Published:** 2018-09-03

**Authors:** Massimiliano Zanin, Alejandro Rodríguez-González, Ernestina Menasalvas Ruiz, David Papo

**Affiliations:** 1 Center for Biomedical Technology, Universidad Politécnica de Madrid, 28223 Pozuelo de Alarcón, 28040 Madrid, Spain; 2 Department of Computer Science, Faculty of Science and Technology, Universidade Nova de Lisboa, 2829-516 Lisboa, Portugal; 3 SCALab UMR CNRS 9193, University of Lille, 59800 Villeneuve d’Ascq, France

**Keywords:** time irreversibility, permutation entropy, visibility graphs, efficient market hypothesis

## Abstract

Time irreversibility, i.e., the lack of invariance of the statistical properties of a system under time reversal, is a fundamental property of all systems operating out of equilibrium. Time reversal symmetry is associated with important statistical and physical properties and is related to the predictability of the system generating the time series. Over the past fifteen years, various methods to quantify time irreversibility in time series have been proposed, but these can be computationally expensive. Here, we propose a new method, based on permutation entropy, which is essentially parameter-free, temporally local, yields straightforward statistical tests, and has fast convergence properties. We apply this method to the study of financial time series, showing that stocks and indices present a rich irreversibility dynamics. We illustrate the comparative methodological advantages of our method with respect to a recently proposed method based on visibility graphs, and discuss the implications of our results for financial data analysis and interpretation.

## 1. Introduction

Time irreversibility is the lack of invariance of the statistical properties of a signal under the operation of time reversal. In other words, consider a time series describing the evolution of a system, x(t) with t∈[0,T] and its time reversal, i.e., the time series that would have been obtained had the system evolved in the opposite direction, or $x^{t.r.}\left(t\right)=x\left(T-t\right)$. Irreversibility means that it is possible to find a characteristic that differs in the forward and backward versions, i.e., a function *f* calculated over the two time series such that $f\left(x^{t.r.}\right)\neq f\left(x\right)$; in other words, the observer can distinguish the forward, from the backward version of a given process. Note that the above definition does not impose any restriction on *f*.

Irreversibility can be due to the presence of memory, which acts as a hidden dissipative external force in a process [1] while the presence of noise results in a loss of irreversibility [2]. Thus, estimating the degree of irreversibility of a time series implicitly quantifies the degree of nonlinear dependences (memory), and, therefore, the degree of time series predictability. Importantly, since linear Gaussian random processes and static nonlinear transformations of such processes are reversible, significant time irreversibility excludes Gaussian linear processes as models for the generating dynamics, implying instead nonlinear dynamics, non-Gaussian (linear or nonlinear), or linear autoregressive moving average (ARMA) models as possible generative processes [3,4,5].

The mere statistics of observed time series allows extracting information on the physics of the system under study. In particular, time reversal asymmetry provides information about the entropy production of the physical mechanism generating the series, even when the details of the underlying generating system are unknown [6]. Various methods to quantify time reversibility have been proposed [7,8,9,10,11] and applied to the study of both biological [12,13] and financial systems [2,14,15,16,17,18,19,20,21].

Here, we introduce a new method, based on permutation entropy [22,23], to evaluate irreversibility of time series at various temporal scales. With respect to existing methods, the proposed one presents various advantages: (1) it has no free parameters other than the embedding dimension of the permutation entropy; (2) similar to visibility graph methods [9], it is temporally local, and therefore allows assessing fluctuations; (3) assessing significance is straightforward, and does not rely on scaling arguments as in visibility graph methods; and (4) it has a convergence speed advantage over visibility graph methods.

We first illustrate our method by evaluating the time irreversibility of a set of simple dynamical models, including stochastic models and chaotic dynamical systems, for which such property has theoretically been studied. We further show how the proposed approach can help elucidate the complex irreversibility dynamics of financial time series, representing 30 major European stocks and 12 world indices.

The time-reversal properties of financial time series allow testing the so-called efficient market hypothesis (EMH) [20]. The EMH asserts that financial markets are efficient with respect to an information set, i.e., that stocks incorporate all publicly available information useful in evaluating their prices and no single market agent can consistently outperform the market with information based trading [24]. Importantly, efficiency is related to the amount of information available to predict future market prices, with lower efficiency corresponding to higher residual predictive information in the past sequence of stock prices [25]. The stringency of EMH’s requirements suggests that no real market can ever be efficient stricto sensu [26] and that EMH should not be approached as an all-or-nothing property [27]. Various empirical studies have then undertaken to quantify the extent to which the EMH holds and, as a result to identify the sort of process governing market behaviour [27,28,29,30]. While financial series have been found to generally be time irreversible [2,14,20,31], it is possible to discriminate different degrees of such property. For instance, some stocks have been found to be more irreversible than others [11]. Likewise, emerging markets have been shown to be more time irreversible than developed ones, lending support to the relationship between efficiency and irreversibility [32].

We show that stocks’ and indices’ time series present a rich dynamics in terms of irreversibility. Specifically, while some time series may globally be reversible, they can become irreversible at specific temporal resolutions, i.e., when windows of specific length are considered. Additionally, such irreversibility may appear in a temporal localised way, suggesting that the dynamics of the element was somehow perturbed at that time.

The remainder of the paper is organised as follows. Firstly, the proposed method is described in Section 2; we also include a brief overview of the visibility graph (Section 2.3) and of the Markov chain (Section 2.4) approaches, as they are used to benchmark our solution. We then validate the permutation patterns’ method in synthetic (Section 3) and financial (Section 4) time series. Some conclusions are finally drawn in Section 5.

## 2. Assessing Time Series Reversibility

### 2.1. Permutation Patterns

The idea of analysing the permutation patterns present in a time series was initially introduced by Bandt and Pompe [22] to provide researchers with a simple and efficient tool to characterise the complexity of the dynamics of real systems. With respect to other approaches, as entropies, fractal dimensions, or Lyapunov exponents, it presents the advantage of being independent from any arbitrary thresholds or binning procedures [23]. For the sake of completeness, we here briefly review the process of calculating these permutation patterns.

Given a time series $X=\{x_{t}\}$, with t=1…N, this is usually divided in overlapping regions of length *D*, such that:

(1) $$s\to (x_{s},x_{s+\tau },\ldots ,x_{s+\tau (D-2)},x_{s+\tau (D-1)}).$$

*D* is called the *embedding dimension*, and controls the quantity of information included in each region, while τ is the embedding delay. *s* further controls the beginning of each region, and thus the degree of overlap between regions. Without loss of generality, in the following, we consider D=3 and τ=1.

The second step involves associating an ordinal pattern to each region. Values are sorted in increasing order, and the ordinal pattern corresponding to the required permutation is saved for further analysis. In other words, the permutation $\pi =(r_{0},r_{1},\ldots ,r_{D-1})$ of (0,1,…,D−1) is defined to fulfil:

(2) $$x_{s+r_{0}}\leq x_{s+r_{1}}\leq \ldots \leq x_{s+r_{D-2}}\leq x_{s+r_{D-1}}.$$

To illustrate, suppose a time series X=(3,2,6,4,8). As D=3, the first region would include the values (3,2,6), and the order required for sorting them is (1,0,2)—that is, the second value is the smallest, followed by the first and by the last. Similarly, the second region (2,6,4) is associated with the pattern (0,2,1); and the third region (6,4,8) with (1,0,2).

### 2.2. Time Reversibility of Permutation Patterns

After estimating all the permutation patterns in a time series, we analyse their frequency of appearance, taking into account a time reversal process.

The total number of permutation patterns that may appear is given by D!. These patterns can be paired together, such that each pattern composing a pair is the time reversal of the other. For instance, for D=3, six patterns are generated, which can pairwise be related as:

(3) $$\begin{array}{c} \left(0,1,2\right)\overset{t.r.}{↔}\left(2,1,0\right) \end{array}$$

(4) $$\begin{array}{c} \left(1,0,2\right)\overset{t.r.}{↔}\left(2,0,1\right) \end{array}$$

(5) $$\begin{array}{c} \left(1,2,0\right)\overset{t.r.}{↔}\left(0,2,1\right), \end{array}$$

with $\overset{t.r.}{↔}$ representing a time reversal transformation.

To clarify this idea, let us consider the simple example of a time series resembling a sawtooth, X=(1,2,3,1,2,3,1). The series is stationary, as the average oscillates around 2.0, and five permutation patterns of side D=3 can be extracted: (0,1,2), (1,2,0), (2,0,1), (0,1,2) and (1,2,0). It can be observed that the system has non-trivial dynamics, as it always increments in two consecutive steps at a time—hence, the upward pattern (0,1,2). Let us now consider the time reversed series, i.e., the same series observed from the end to the beginning: X=(1,3,2,1,3,2,1). The new (time reversed) permutation patterns are (0,2,1), (2,1,0), (1,0,2), (0,2,1) and (2,1,0). As it should be expected, the new time series can only diminish through the (2,1,0) permutation pattern—which is, of course, the time reversal equivalent of (0,1,2). Note that this allows us to conclude that the time series *X* is irreversible. Specifically, let us suppose that we observe a realisation of *X* with two consecutive increasing values, i.e., (0,1,2); as this can only appear in the original time series, and not in the time reversed one, we can then be certain about the time directionality. In a similar fashion, if we observe the pattern (2,1,0), we can conclude that we are observing the time reversed series.

This can further be generalised: a time series will be reversible if and only if all permutation patterns composing the previous pairs appear with approximatively the same frequency. In the previous example, we can observe that the probability of (0,1,2) and (2,1,0) in the original time series are different—respectively, $p_{\left(0,1,2\right)}=2/5$ and $p_{\left(2,1,0\right)}=0/5$. Once again, *X* is then expected to be irreversible. This idea constitutes the basis of the reversibility statistical test described below.

The value of D=3 has here been chosen for the sake of clarity. While in principle larger values of *D* may yield a richer description of the dynamics, this also results in the need of longer time series to reach statistically significant results—both topics will be further discussed in the conclusions.

The previously defined pattern pairs and their frequency of appearance can be analysed in two ways: in terms of the magnitude of the irreversibility, through the Kullback–Leibler divergence [33], and in terms of its statistical significance, through a binomial test.

The irreversibility magnitude can be quantified by comparing two probability distributions, one represented by the probability of all patterns appearing in the direct (or original) time series, and a second one with the probabilities for the time-reversed time series. Following the previous example for D=3, the first distribution is composed of the frequencies of patterns $P_{d}=\left[p_{\left(0,1,2\right)},p_{\left(2,1,0\right)},p_{\left(1,0,2\right)},p_{\left(2,0,1\right)},p_{\left(1,2,0\right)},p_{\left(0,2,1\right)}\right]$. As for the second distribution, it can be calculated by actually reversing the time series, or more simply by using the previous time reversal transformations—i.e., by considering the distribution $P_{r}=\left[p_{\left(2,1,0\right)},p_{\left(0,1,2\right)},p_{\left(2,0,1\right)},p_{\left(1,0,2\right)},p_{\left(0,2,1\right)},p_{\left(1,2,0\right)}\right]$. The difference between both distributions can then be estimated through the Kullback–Leibler divergence:

(6) $$D_{KL}=\sum_{i=1}^{D!}P_{d}\left(i\right)\log\frac{P_{d}\left(i\right)}{P_{r}\left(i\right)}.$$

If the time series is perfectly reversible, the probabilities associated to patterns forming a pair should be the same, thus yielding a $D_{KL}\approx 0$. On the other hand, the higher the value of $D_{KL}$, the more irreversible the time series is. Note that $D_{KL}$ is not the only possibility for comparing the two distributions, being the Jensen–Shannon divergence a good alternative [34]. While the latter presents the advantage of being symmetric, the former is commonly used in statistical physics [9,35]. Additionally, it has to be noted that Equation (6) diverges when one or more permutation patterns are forbidden, i.e., their frequency is zero. This may happen when the time series under analysis is trivially irreversible, and possibly non-stationary. This is for instance the case of the ramp function previously described; when i=0 in Equation (6), the argument of the logarithm becomes $p_{\left(0,1,2\right)}/p_{\left(2,1,0\right)}\;=\;2/0$, and thus $D_{KL}\to \infty$. While this clearly indicates that the time series is (infinitely) irreversible, the divergence of $D_{KL}$ may make subsequent calculations more complicated. This can easily be solved by adding a very small value to all probabilities, i.e.,

(7) $$D_{KL}=\sum_{i=1}^{D!}P_{d}\left(i\right)\log\frac{P_{d}\left(i\right)+\epsilon }{P_{r}\left(i\right)+\epsilon },$$

such that $\epsilon \ll \min P_{d}$ and $\epsilon \ll \min P_{r}$. This situation is nevertheless seldom encountered in real time series, provided their length is large enough.

If the Kullback–Leibler divergence tells us the magnitude of the irreversibility of a time series, it yields little information about the statistical significance of the value. This problem can be solved by levering on the binomial nature of the patterns composing a pair. Specifically, if the time series is reversible, the number of times the two permutation patterns forming a pair appear should not statistically be different. Following the previous example, let us denote by $n_{\left(0,1,2\right)}$ and $n_{\left(2,1,0\right)}$, respectively, the number of times the patterns (0,1,2) and (2,1,0) have appeared; and let us define:

(8) $$p=\frac{n_{\left(0,1,2\right)}}{n_{\left(0,1,2\right)}+n_{\left(2,1,0\right)}}.$$

The time series is not reversible if we can reject the null hypothesis that p=0.5 in a two-sided binomial test. Note that the test should be repeated for all pairs of permutation patterns—three times in the case of D=3.

One final discussion should here be added on the relationship between irreversibility and stationarity, and how such relationship affects the proposed methodology. On the one hand, it is intuitive that a non-stationary process must also be irreversible—as a net change from state *a* to state *b* necessarily implies a time direction. Time irreversibility has therefore normally been assessed only in the presence of stationarity. On the other hand, it has recently been proposed that reversibility can be assessed even in non-stationary systems, by moving from a qualitative to a quantitative metric [35]. In the case of the methodology here proposed, the degree of irreversibility of a time series can be assessed by the magnitude of $D_{KL}$ (or of a Jensen–Shannon divergence), provided no permutation pattern is forbidden, i.e., $P_{r}\left(i\right)>0$ for all *i*. This quantitative aspect will be further explored in Section 4.

### 2.3. Directed Horizontal Visibility Graphs

One of the most recent and efficient ways of assessing the irreversibility of a time series is through the so-called directed Horizontal Visibility Graphs (dHVG). In what follows, this method is used for benchmark purposes, and, for the sake of completeness, is here briefly introduced.

From a general point of view, dHVG belong to a family of methods that map a time series into nodes of a network, based on geometric criteria [36,37]. In all of these methods, a complex network [38] is created, whose nodes correspond to the individual data of the time series; pairs of nodes are then connected when they fulfil some geometrical rule, usually based on whether one value can “see” the other one. In the specific case of dHVG, two nodes are connected if the line connecting both values is not obstructed by another intermediate point [37]. Mathematically, given two nodes *i* and *j*, a link is created if:

(9) $$x_{i},x_{j}>x_{n},\forall n|i<n<j,$$

being $x_{i}$ the element of the time series mapped into node *i*.

The resulting network can then be analysed using the wide set of tools provided by complex networks theory [39]. Of relevance for this work, the irreversibility of a time series can be assessed by comparing the distributions of in- and out-degrees (i.e., respectively the number of links arriving to and departing from a given node), and by calculating a Kullback–Leibler divergence [9,11]. Note that the in-degree of a node becomes its out-degree under a time reversal transformation. Therefore, for reversibility to holds both distributions ought to be equal, and the corresponding Kullback–Leibler divergence should converge to zero. For more details on the dHVG approach and the assessment of irreversibility, we refer the reader to the following studies [9,11,37].

### 2.4. Markov Chain Approach

We finally consider a classical method for detecting time series irreversibility, based on the representation of the underlying system as a Markov chain. In the case of a Markov chain with a transition matrix $P_{i,j}$ and steady-state distributions $\pi_{i}$, time symmetry implies $\pi_{i}P_{i,j}=\pi_{j}P_{j,i}$; a time series is then reversible if and only if $P_{i,j}=P_{j,i}$, for all is and j≠i [40]. We use this property to construct a simple test, which requires: (i) binning the elements of the original time series into a set of bins (note that the number of bins is a parameter of the method); (ii) calculate the transition matrix $P_{i,j}$; and (iii) perform a binomial statistical test on each pair (i,j), with j≠i, to test the hypothesis that $P_{i,j}=P_{j,i}$.

## 3. Validation with Synthetic Time Series

We validate the permutation patterns approach to irreversibility assessment, and compare it with the visibility graph one, through the application to a set of synthetic time series whose reversible or irreversible nature has already been studied theoretically. These are:

- Two reversible stochastic processes, namely a time series of values drawn from a Gaussian distribution N(0,1), and an Ornstein–Uhlenbeck process, a mean-reverting linear Gaussian process T [41].
- Two dissipative chaotic maps, respectively, a logistic map (defined as $x_{n+1}=ax_{n}\left(1-x_{n}\right)$, with a=4.0) and a Henon map ($x_{n+1}=1+y_{n}-ax_{t}^{2}$, $y_{n+1}=bx_{t}$, with a=1.4 and b=0.3). Dissipative systems are by definition irreversible [42].
- The Arnold Cat map, and example of a conservative chaotic map ($x_{n+1}=x_{n}+y_{n}\;\mathrm{mod}\;\left(1\right),\;y_{n+1}=x_{n}+2y_{n}\;\mathrm{mod}\;\left(1\right)$. The analysed time series corresponds to the evolution of the *x* variable.
- The Lorenz chaotic system, defined as $\dot{x}=\sigma \left(y-x\right)$, $\dot{y}=x\left(\rho -z\right)-y$, and $\dot{z}=xy-\beta z$ (with ρ=28, σ=10 and β=8/3, integration step of dt=0.01). Unless otherwise stated, the analysed time series corresponds to the evolution of the *x* variable.
- Time series generated through an Autoregressive Conditional Heteroskedasticity (ARCH) model [43] defined as $x_{t}=\sigma_{t}z_{t}$, with $\sigma_{t}^{2}=\alpha^{*}\left(1+\sum_{i=1}^{3}2^{-i}x_{t-i}^{2}\right)$ and $z_{t}$ being independent random numbers drawn from an uniform distribution U(0,1). Note that $\alpha^{*}$ is a parameter controlling the strength of the time dependence between present and past values of *x*, and hence its irreversibility.
- Time series generated through a Generalised Autoregressive Conditional Heteroskedasticity (GARCH) model [44] defined as $x_{t}=\sigma_{t}z_{t}$, with $\sigma_{t}^{2}=\alpha^{*}\left(1+\sum_{i=1}^{3}2^{-i}x_{t-i}^{2}+\sum_{i=1}^{3}2^{-i}\sigma_{t-i}^{2}\right)$ and $z_{t}$ being independent random numbers drawn from an uniform distribution U(0,1). Note that the difference with respect to the ARCH model resides in the fact that here σ depends directly on its past. As in the previous case, $\alpha^{*}$ is controlling the time irreversibility of the model.

**Figure 1:**
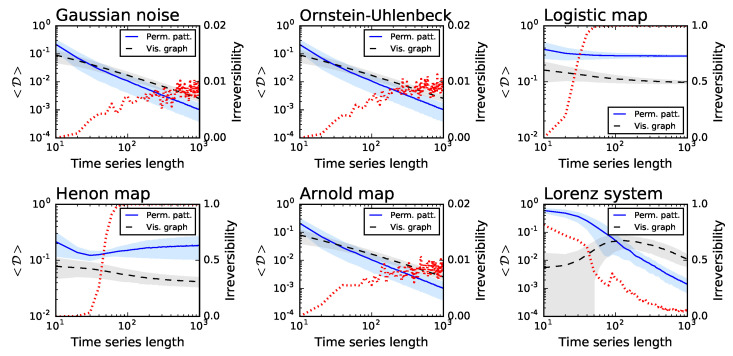
Irreversibility analysis of several synthetic dynamical models, as a function of the time series length. From left to right, top to bottom, the six panels represent Gaussian noise, an Ornstein–Uhlenbeck process, logistic, Henon and Arnold maps, and a Lorenz oscillator—see main text for details and parameters. In the left Y axis, the blue solid and black dashed lines, respectively, represent the average Kullback–Leibler divergence obtained by the permutation patterns and the visibility graph approach—note the blue and grey bands, depicting one standard deviation. On the right Y axis, the dotted red line indicates the fraction of simulations in which the time series is irreversible in a statistical significant way, with α=0.01.

**Figure 2:**
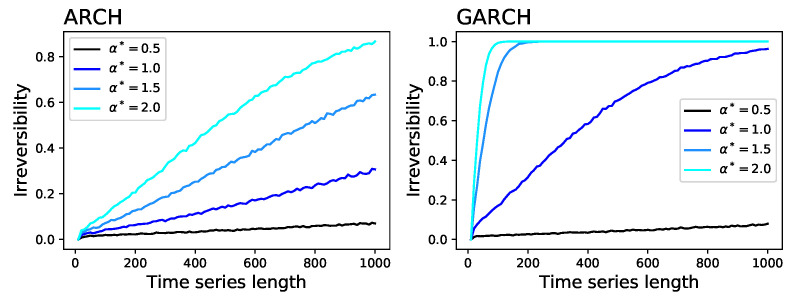
Irreversibility of time series generated by: ARCH model (**Left**); and GARCH model (**Right**). Each line indicates the fraction of simulations in which the time series is irreversible in a statistical significant way, with α=0.01, as a function of the time series length and of the value of $\alpha^{*}$ (see main text for definitions).

**Figure 3:**
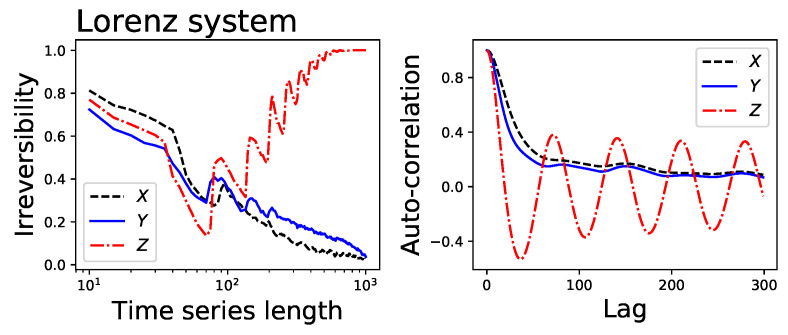
(**Left**) Fraction of irreversible time series yielded by a Lorenz chaotic system, as a function of the time series length, where black (dashed), blue (solid) and red (dash-dot) lines correspond respectively to the *X*, *Y* and *Z* channels of the system; and (**Right**) autocorrelation of the same three time series.

For each of them, Figure 1 reports: (i) the average divergence D yielded by the permutation patterns (blue line and one standard deviation band) and the visibility graph (black line and band) approaches; and (ii) the fraction of times the time series is detected as irreversible by the permutation patterns approach in a statistical significant way (red dotted line, right Y axis, significance α=0.01). The irreversibility of the time series created by the ARCH and GARCH models is reported in Figure 2, as a function of the parameter $\alpha^{*}$. The two examples of stochastic processes and the Arnold map are recognised as irreversible in less than 1% of the realisations—as expected from the choice of a statistical significance level of α=0.01. On the other hand, the irreversibility frequency rapidly converges to one for the two dissipative chaotic maps, which are known to be irreversible [42]. Intermediate results can be observed in the case of the ARCH and GARCH models, for which a robust irreversibility is detected in the case of long time series and high values of $\alpha^{*}$. Finally, a special situation can be observed for the Lorenz system: while its time series are mostly irreversible at short temporal scales, they become highly reversible when sufficiently long time windows are considered. To understand if such behaviour is a general property of the system, Figure 3 (Left) reports the evolution of the irreversibility as a function of time series length, for the three channels of the Lorenz system. While the *X* and *Y* channels have a similar dynamics, the *Z* one is substantially different: first it is completely irreversible over long time scales, and second, the evolution of the irreversibility is not monotonic, with a minimum around 70 and a peak every 60 time points. This abnormal behaviour for the *Z* time series is possibly due to its dynamics, which is well known to differ from those of the *X* and *Y* channels in terms of Lyapunov exponent [45] and autocorrelation (see Figure 3, Right).

**Figure 4:**
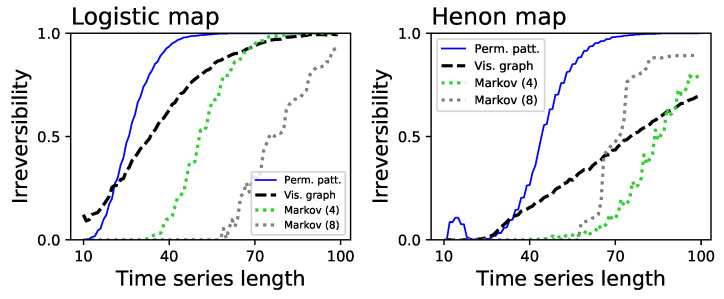
Analysis of the time series length required to reach a consistent irreversibility assessment. Both panels depict the fraction of times the permutation patterns (blue solid lines), the visibility graph algorithms (black dashed lines) and the Markov chain method (dotted lines) detect a statistically significant irreversibility, as a function of the time series length: (**Left**) logistic map; and (**Right**) Henon map.

Figure 1 further suggests that the permutation patterns approach to irreversibility can be more sensitive than the visibility graph one—note that the <D> blue lines usually have a steeper slope, and converge faster than the black ones. Figure 4 depicts the fraction of times the three considered methods detect that the underlying time series is irreversible in a statistical significant way (α=0.01), for very short time series lengths and for the two systems that were detected as irreversible (i.e., respectively, the Logistic and Henon maps). Note that, to calculate the statistical significance of the divergence yielded by the visibility graph approach, this has been compared with the ones obtained from randomly shuffled versions of the time series, and the probability of finding a larger D in the random realisations expressed as a *p*-value. Figure 4 indicates that the permutation pattern approach requires shorter time series to reach a consistent output, something that is particularly conspicuous in the case of the Henon map. Additionally, these results highlight the benefit associated to parameter-free methods. Specifically, the Markov chain method has been tested with two different numbers of bins, respectively 4 (green dotted lines) and 8 (grey dotted lines), yielding different results depending on the underlying dynamics. The fact that the proposed methodology required no parameter estimation or tuning thus becomes an important practical advantage.

**Figure 5:**
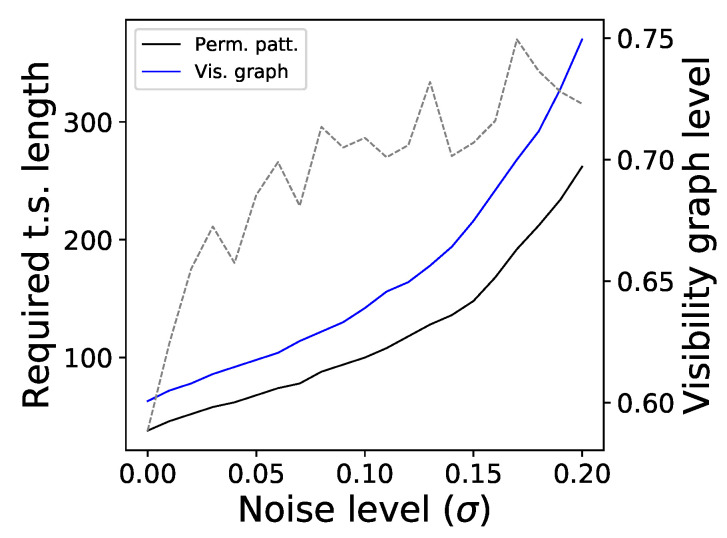
Resilience to noise. The two solid lines (left Y axis) depict the evolution of the time series length required to reach a 90% detection of irreversibility for the logistic map, according to the permutation patterns approach (black) and the visibility graph one (blue), as a function of the level noise. The dashed line (right Y axis) indicates the fraction of times the visibility graph method is detecting an irreversibility, when the permutation patterns method has reached a 90%.

Finally, Figure 5 explores the resilience of the proposed method with respect to the presence of noise. Specifically, we consider the previously described logistic map, and added a Gaussian noise:

(10) $$x_{n+1}=ax_{n}\left(1-x_{n}\right)+\sigma \xi ,$$

with a=4.0 and ξ being independent random numbers drawn from a Gaussian distribution N(0,1). Note that noise is inherently reversible, and therefore its presence is expected to mask the irreversibility of the logistic map. We then measure the minimum time series length that allows to detect the irreversibility of the system the 90% of the times, and plot this as a function of the noise level σ. The two solid lines in Figure 5 report the results, and indicate that the permutation patterns approach is more resilient than the visibility graph one.

Taken together, the numerical experiments carried out on synthetic time series indicate that the permutation patterns approach is comparable to the visibility graph one in assessing irreversibility. The former is nevertheless more sensitive, as it relies more on local patterns (of dimension *D*), and more resilient to noise, thus more suitable for the analysis of short time series. We take advantage of this in Section 4 by analysing the temporal evolution of the irreversibility of real time series. Finally, the local nature of the permutation patterns approach makes it extremely computationally efficient—with a computational cost that scales linearly with the number of data points, as opposed to the quadratic growth of the visibility graph approach.

## 4. Application to Financial Time Series

To further validate the proposed methodology, we assess the irreversibility of several financial time series. These can be thought of as relatively short realisations of complex stochastic processes whose dynamics is richer than most of the generated time series, and their characteristics (including reversibility) can change over time. Dynamical repertoire richness and time series shortness are two desirable aspects from a validation view-point. As previously introduced, if financial time series were shown to be irreversible, i.e., if some permutation patterns were favoured over their corresponding time-reversed counterparts, this would disprove the efficient market hypothesis (EMH) [20], as the asymmetry would be associated with information with which to improve the prediction of future prices.

We consider two sets of time series representing the daily evolution of, on the one hand, the Top-30 European stocks by capitalisation; and, on the other hand, of 12 representative world stock market indices. Table 1 and Table 2 report the two full lists, along with some basic characteristics. Both sets of time series have been obtained through Yahoo Finance, and include data from 1 January 2008 to 1 January 2018—note that the actual number of data points may differ, e.g., due to local bank holidays. To ensure the stationarity of all time series, the original values $X_{t}$ have been transformed to ${\hat{X}}_{t}=\log_{2}X_{t+1}/X_{t}$. The resulting series $\hat{X}$ have been tested through an Augmented Dickey–Fuller unit root test [46], and for all of them the presence of a unit root was rejected in a statistically significant way (the larger *p*-value being $2.48\times 10^{-14}$ for the BNP.PA stock).

Each time series was analysed in three different ways. The first one entails estimating global irreversibility, i.e., taking into account the whole time series. This corresponds to the irreversibility of the system, under the assumption that such property is stationary, or to the assessment of the average irreversibility. Three stocks and four indices resulted irreversible: BBVA.MC, ENEL.MI, and G.MI; and DJI, GDAXI, GSPC and IXIC, respectively. This indicates that markets have preferred ways (or patterns) when rallying up- or downwards, and are therefore strictly not efficient. It is also interesting to observe that irreversibility is more frequent in indices (four out of twelve) than in individual stocks; this may suggest that irreversibility is a collective (or emergent) phenomenon, which is difficult to see in the dynamics of individual elements, but shows up when considering groups of them.

**Figure 6:**
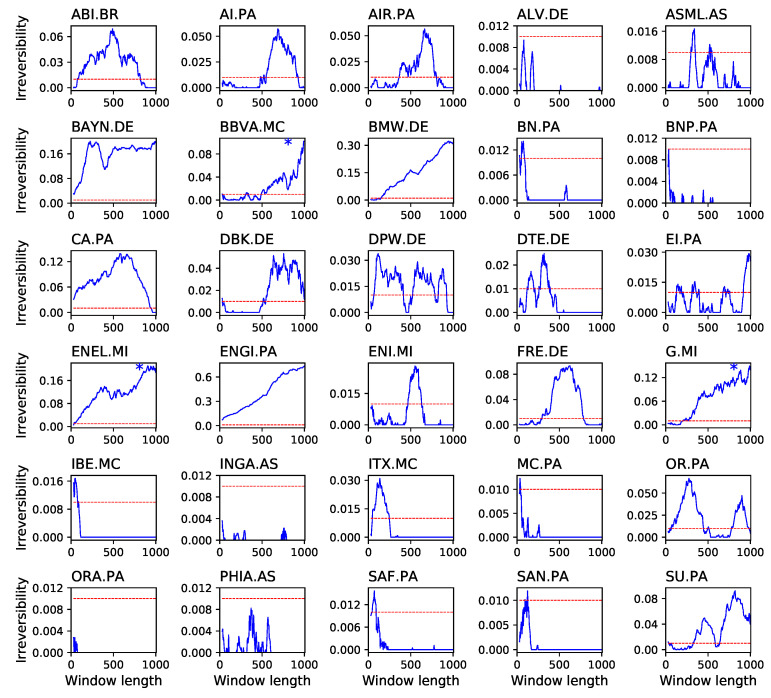
Reversibility of the 30 biggest European stocks by capitalization. The solid line of each panel depicts the fraction of windows in which the absence of reversibility was statistically significant (α=0.01, Y axes), as a function of the window size in days (X axes). The horizontal dashed line represents the significance level of 0.01. An asterisk in the top right corner of a panel indicates that the stock is reversible when considering the whole time series.

**Figure 7:**
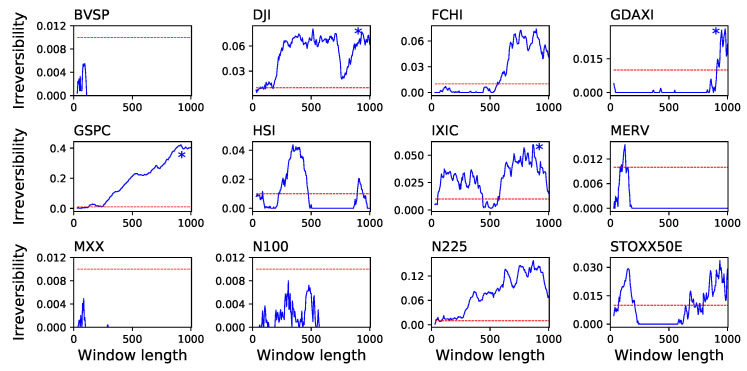
Reversibility of 12 market indices. The solid line of each panel depicts the fraction of windows in which the absence of reversibility was statistically significant (α=0.01, Y axes), as a function of the window size in days (X axes). The meaning of the horizontal dashed lines and of the asterisks is the same as in Figure 6.

Even when the complete time series is reversible, it is possible to find shorter sub-windows which are not reversible in a statistically significant way. Thus, it may happen that time series are globally reversible, but locally irreversible. We explore this possibility in a second analysis, in which we extract all possible sub-windows of a given length from each time series, and calculate their average irreversibility. Note that this allows estimating irreversibility as a function of the time window length, and thus the relationship between irreversibility and time scales. In other words, this second approach enables to study the local vs. global nature of irreversibility. Results of this analysis, in terms of the fraction of windows yielding a statistically significant irreversibility (α=0.01) as a function of the window length, are presented in Figure 6 and Figure 7. Three general ideas can be drawn from these results. First, many time series that are globally reversible display noisy results, with very low irreversibility probabilities, and usually around or below the significance threshold. Secondly, those time series that are globally irreversible gain such properties at relatively long time scales—the evolution of the fraction of irreversible windows constantly increases with the window size. Specifically, when the average irreversibility of each time series is calculated for window lengths comprised between (0,500) and (500,1000), the resulting correlation coefficient is of 0.92612 for the 30 stocks and of 0.879552 for the 12 indices. This seems to indicate that the time series are highly noisy, and therefore that long time windows are required to reach a stable result—as previously shown in Figure 5. Finally, some time series, which are globally reversible, can contain irreversible windows with a significant probability; it thus seem that, for those time series, irreversibility is a property confined to some specific time scales. This is the case, for instance, of BAYN.DE (maximum of 20.12% for lengths of 225) or CA.PA (13.89% at 575).

**Figure 8:**
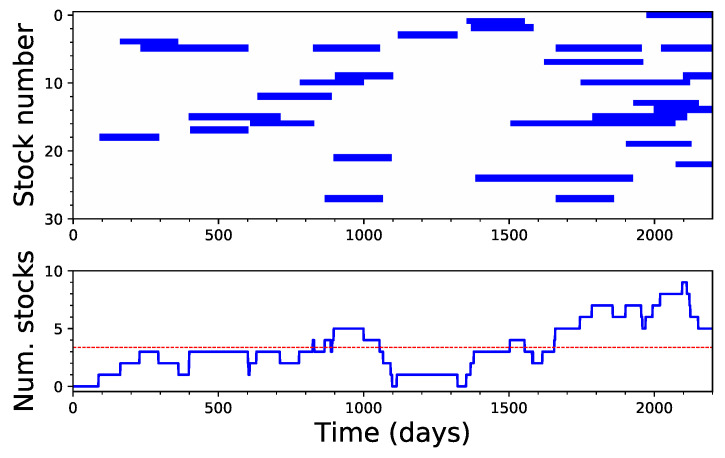
Analysis of the synchronicity between irreversible windows: (**Top**) the time intervals when each stock time series is detected as irreversible, using windows of 200 data points; and (**Bottom**) the evolution of the number of stocks that were irreversible at the same time. The dashed red line represents the expected number of irreversible stocks under the assumption of independence.

Given that irreversibility is, in many cases, a localised effect, we finally checked whether different stocks present a synchronised dynamics, i.e., if different stocks tend to become irreversible at the same time. Figure 8 presents a time map of the irreversibility of the 30 analysed stocks, when considering windows of 200 data points. While irreversibility seems to be slightly more probable at the end of the considered period, deviations from the expected value are not enough to support the hypothesis of synchronous dynamics.

## 5. Discussion and Conclusions

**Figure 9:**
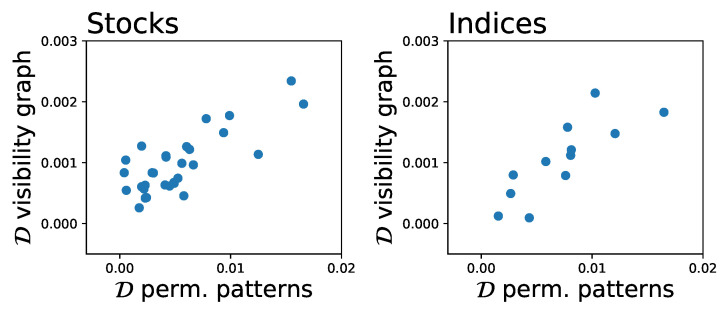
Analysis of the similarity of the irreversibility, as yielded by the proposed method and by the visibility graph approach: (**Left**) stocks time series; and (**Right**) indices time series.

We proposed a new method to quantify irreversibility in time series based on permutation entropy. We tested our method on synthetic time series from various processes with known irreversibility properties and on financial time series of stock prices and indices. For synthetic time series, the results from our method are consistent with known irreversibility properties of the respective time series. Remarkably, particularly for the Lorenz system, the method could detect non-trivial irreversibility dynamics. Our results also show that, while most financial time series are globally reversible, the proposed method highlighted an interesting dynamics, with time windows in which the dynamics was significantly irreversible. While the results from the permutation entropy-base method were in line with those obtained with the dHVG-based method (see Figure 9), the former method compared favourably in terms of convergence speed, indicating that it can be more suitable for relatively short time series. Additionally, the proposed method is able to better handle singular situations, provided the modified version of Equation (7) is used. For instance, it is able to detect the extreme irreversibility of a ramp function; on the contrary, for such time series, the dHVG-based method yields regular networks with a constant degree of 1, as in both directions each value can only “see” the following one, thus returning a D of zero and wrongly suggesting a perfect reversibility.

Our results with synthetic time series are consistent with theoretical results, indicating that the proposed method correctly identifies the underlying process. On the other hand, some results for financial time series are somehow surprising. In particular, our method returned higher irreversibility for some markets previously known to be among the most efficient ones (see Figure 7). These results were in good agreement with those obtained using dHVGs. Insofar as the presence of irreversibility may be associated with violation of the EMH, our results suggest that permutation entropy-based irreversibility and dHVGs may capture a dynamical feature that differs from standard measures of market efficiency. Further investigations will be needed to clarify the reasons for this discrepancy. Additionally, it is important to exercise caution against too literal an interpretation of financial systems’ random walk in the same thermodynamic terms (viz., friction and dissipation) as the original Langevin equation [20]. Finally, it is worth pointing out that various studies [2,20,32] have suggested that a complete picture of irreversibility in financial time series should be based on a multiscale analysis, as the relationship among scales may contain important features ultimately determining time irreversibility [20]. On the other hand, analyses at given scales, as in the present study, should be interpreted in terms of irreversibility at that particular scale.

One final note should be made on the choice of the embedding dimension *D*, which we set to D=3 in this study. Using higher values of *D* increases the richness with which the dynamics of the system is captured—see for an example [47]. In addition, it has been shown that the permutation entropy (a closely related concept) is an approximation that converges to the true entropy rate of the system in the limit of increasing embedding dimension. It is thus logical to expect a similar behaviour for the proposed measure of reversibility, which may converge to a real value for large values of *D*. It is nevertheless important to take into account that increasing *D* also comes with several disadvantages. First, obtaining reliable statistics on the appearance of the permutation patterns and reducing the influence of random fluctuations requires longer time series—as a rule of thumb, it is usually recommended to have time series of length of at least (D+1)! [48]. This limits the resolution of the irreversibility analysis, and precludes detecting interesting phenomena at short time scales (as shown in Figure 3). Second, although, from a theoretical point of view, nothing precludes the use of higher embedding dimensions in the methodology proposed in this study, the computational cost scales exponentially with the embedding dimension—a limitation that may become serious when analysing large datasets as in some real-time applications.

## Figures and Tables

**Table 1:** List of the 30 considered stocks.

Stock Code	Name	Country	Capitalisation
ABI.BR	Anheuser Busch Inbev NV	Belgium	182.039 B€
AI.PA	Air Liquide	France	46.635 B€
AIR.PA	Airbus SE	France	72.22 B€
ALV.DE	Allianz SE	Germany	91.67 B€
ASML.AS	ASML Holding N.V.	Netherlands	71.596 B€
BAYN.DE	Bayer AG	Germany	87.425 B€
BBVA.MC	Banco Bilbao Vizcaya Argentaria, S.A.	Spain	49.919 B€
BMW.DE	Bayerische Motoren Werke AG	Germany	62.545 B€
BN.PA	Danone SA	France	44.386 B€
BNP.PA	BNP Paribas SA	France	84.307 B€
CA.PA	Carrefour SA	France	14.13 B€
DBK.DE	Deutsche Bank AG	Germany	32.651 B€
DPW.DE	Deutsche Post AG	Germany	48.763 B€
DTE.DE	Deutsche Telekom AG	Germany	69.937 B€
EI.PA	Essilor International SA	France	24.22 B€
ENEL.MI	Enel SpA	Italy	53.528 B€
ENGI.PA	ENGIE SA	France	34.648 B€
ENI.MI	Eni S.p.A.	Italy	53.801 B€
FRE.DE	Fresenius SE & Co. KGaA	Germany	37.235 B€
G.MI	Assicurazioni Generali S.p.A.	Italy	25.281 B€
IBE.MC	Iberdrola, S.A.	Spain	42.207 B€
INGA.AS	ING Groep N.V.	Netherlands	64.689 B€
ITX.MC	Industria de Diseño Textil, S.A.	Spain	89.425 B€
MC.PA	LVMH Moët Hennessy Louis Vuitton S.E.	France	121.994 B€
OR.PA	L’Oréal S.A.	France	102.244 B€
ORA.PA	Orange S.A.	France	39.275 B€
PHIA.AS	Koninklijke Philips N.V.	Netherlands	31.07 B€
SAF.PA	Safran SA	France	37.748 B€
SAN.PA	Sanofi SA	France	87.918 B€
SU.PA	Schneider Electric S.E.	France	42.25 B€

**Table 2:** List of the 12 considered market indices.

Index Code	Name	Country
BVSP	IBOVESPA	Brasil
DJI	Dow Jones Industrial Average	USA
FCHI	CAC 40	France
GDAXI	DAX	Germany
GSPC	S&P 500	USA
HSI	Hang Seng Index	Hong Kong
IXIC	NASDAQ Composite	USA
MERV	MERVAL Buenos Aires	Argentina
MXX	IPC Mexico	Mexico
N100	EURONEXT 100	Europe
N225	Nikkei 225	Japan
STOXX50E	EURO STOXX 50	Europe
